# Role of *GALNT12* in the genetic predisposition to attenuated adenomatous polyposis syndrome

**DOI:** 10.1371/journal.pone.0187312

**Published:** 2017-11-02

**Authors:** Víctor Lorca, Daniel Rueda, Lorena Martín-Morales, Carmen Poves, María Jesús Fernández-Aceñero, Clara Ruiz-Ponte, Patricia Llovet, David Marrupe, Vanesa García-Barberán, Beatriz García-Paredes, Pedro Pérez-Segura, Miguel de la Hoya, Eduardo Díaz-Rubio, Trinidad Caldés, Pilar Garre

**Affiliations:** 1 Laboratorio de Oncología Molecular, CIBERONC, IdISSC, Hospital Clínico San Carlos, Madrid, Spain; 2 Laboratorio de Cáncer Hereditario, Servicio de Bioquímica, i+12, Hospital 12 de Octubre, Madrid, Spain; 3 Servicio de Aparato Digestivo, Hospital Clínico San Carlos, Madrid, Spain; 4 Servicio de Anatomía Patológica, Hospital Clínico San Carlos, Madrid, Spain; 5 Fundación Pública Galega de Medicina Xenómica (FPGMX)-SERGAS, Grupo de Medicina Xenómica-USC, IDIS. Santiago de Compostela, Spain; 6 Servicio de Oncología Médica. Hospital de Móstoles. Madrid. Spain; 7 Servicio de Oncología Médica, CIBERONC, Hospital Clínico San Carlos, Madrid, Spain; Singapore General Hospital, SINGAPORE

## Abstract

The involvement of *GALNT12* in colorectal carcinogenesis has been demonstrated but it is not clear to what extent it is implicated in familial CRC susceptibility. Partially inactivating variant, NM_024642.4:c.907G>A, p.(D303N), has been previously detected in familial CRC and proposed as the causative risk allele. Since phenotypes of the described carrier families showed not only CRC but also a polyp history, we hypothesized that *GALNT12* could be involved in adenoma predisposition and consequently, in hereditary polyposis CRC syndromes. For that purpose, we have screened the *GALNT12* gene in germline DNA from 183 unrelated attenuated polyposis patients. c.907G>A, p.(D303N) was detected in 4 cases (MAF = 1.1%) and no other candidate variants were found. After segregation studies, LOH analyses, glycosylation pattern tests and case-control studies, our results did not support the role of c.907G>A, p.(D303N) as a high-penetrance risk allele for polyposis CRC.

## Introduction

Polypeptide N-acetylgalactosaminyltransferase 12 (GALNT12) is part of a large family of hexosyltransferases involved in the initial steps of mucin-type *O*-glycosylation process [1]. Alterations in the activity of these enzymes lead to aberrant glycosylation, which has been associated with alterations in cell growth, differentiation, transformation, adhesion, metastasis and immune surveillance in cancers [2]. Particularly, GALNT12 is highly expressed in the digestive tract [1] and it is frequently downregulated in colorectal cancer (CRC) [3].

Evidence on the association between *GALNT12* (MIM#610290) and colorectal carcinogenesis had been reported when 6 fully and 1 partially inactivating variants were detected in heterozygosis in the germline DNA of CRC patients, while no inactivating variants were detected in a cohort of cancer-free controls older than 70 [4]. Tumors from the 6 fully inactivating mutation carriers showed aberrant glycosylation patterns of MUC1, main target of GALNT12 in the digestive tract, providing evidence of their involvement in CRC carcinogenesis. However, the variant NM_024642.4:c.907G>A, p.(D303N), which showed a partial activity of 37% compared to the wild type protein was not tested, leaving its role in the genesis of CRC unclear. Later on, c.907G>A, p.(D303N) was detected in three families fulfilling the Bethesda clinical criteria for the identification of individuals at risk for Hereditary nonpolyposis colorectal cancer (HNPCC) along with a high polyp burden [5]. Authors proposed inactivating *GALNT12* alleles, in particular, c.907G>A, p.(D303N), as high-predisposition risk alleles which could explain part of the missing heritability of familial CRC. Recently, no functionally relevant mutations at the *GALNT12* locus were detected in a cohort of CRC type X (families fulfilling the strict Amsterdam clinical criteria for the identification of HNPCC and displaying mismatch-proficient tumors), what discarded this gene as a major predisposition gene for HNPCC [6].

Adenomatous polyposis (AP) is characterized by the development of adenomas along the large intestine and rectum, conferring a high risk of CRC. Therefore, it is a CRC-predisposition syndrome whose risk of cancer depends on the severity of the polyposis. The attenuated forms of adenomatous polyposis (AAP) constitute a large and heterogeneous group in terms of polyposis severity, family history, and genetic predisposition. Around 20% of AAP can be explained by germline mutations in well-known high-penetrance predisposition genes, such as *APC* and *MUTYH* [7] or the newly described *POLE* [8], *POLD1* [8], *NTHL1* [9] and *MSH3* [10]. Nevertheless, the vast majority is still not explained, pointing to the involvement of other unknown high-predisposition genes, polygenic inheritance consisting in the additive effect of low-predisposition alleles or environmental factors.

Given that inactivating *GALNT12* variants were detected in Bethesda families with a high polyp burden [5], we hypothesized that *GALNT12* could account for part of the unexplained AAP susceptibility. Therefore, our aim is to contribute to the clarification of the role of *GALNT12* in the predisposition to familial polyposis CRC through the screening and characterization of *GALNT12* mutations in an unexplained AAP cohort.

## Material and methods

### Study cohort

Inclusion criteria were more than 20 accumulated or 10 synchronic adenomas regardless of age or more than 10 adenomas before the age of 70. Previous detection of candidate variants in any known CRC predisposition gene was established as exclusion criteria.

Germline DNAs from a total of 183 unrelated polyposis cases were included in the study; 127 subjects from Hospital Clínico San Carlos (Madrid), 37 from Hospital 12 de Octubre (Madrid) and 19 from Grupo de Medicina Xenomica-USC (Galicia). Ethical approval was obtained from Hospital Clínico San Carlos’ Ethical Research Committee (approval number: C:P:-14/241-E_BS). A written informed consent was obtained from each participant.

### *GALNT12* mutational screening

The first 164 samples (Madrid) were screened through an NGS Haloplex custom panel (Agilent Technologies, U.S.) of 22 CRC-related predisposition genes sequenced in a MiSeq System (Illumina, U.S.) (Table 1). Data analysis and variant calling were performed with the SureCall software (Agilent Technologies, U.S.). Exome data provided by Grupo de Medicina Xenomica-USC (Galicia) was used to screen for *GALNT12* variants in the remaining 19 samples. The exome enrichment system used was SureSelect Human All Exon v6 (Agilent Technologies) and sequencing was carried out in an Ion Proton System (Thermo Fisher Scientific, U.S.).

**Table 1:** List of CRC predisposition genes included in the NGS custom panel.

GENE	TARGET COVERAGE	GENE	TARGET COVERAGE
APC	99.8%	MSH4	100%
AXIN2	99.7%	MSH6	99.98%
BMP4	100%	MUTYH	100%
BMPR1A	98.82%	NTHL1	100%
ENG	100%	PMS2	99.22%
GALNT12	100%	POLD1	100%
GREM1	100%	POLE	99.68%
MLH1	100%	PTEN	100%
MLH3	99.06%	SCG5	99.87%
MSH2	100%	SMAD4	100%
MSH3	100%	STK11	100%

All coding regions, intron-exon boundaries and UTR regions of the listed genes were included in the custom panel

**Fig 1:**
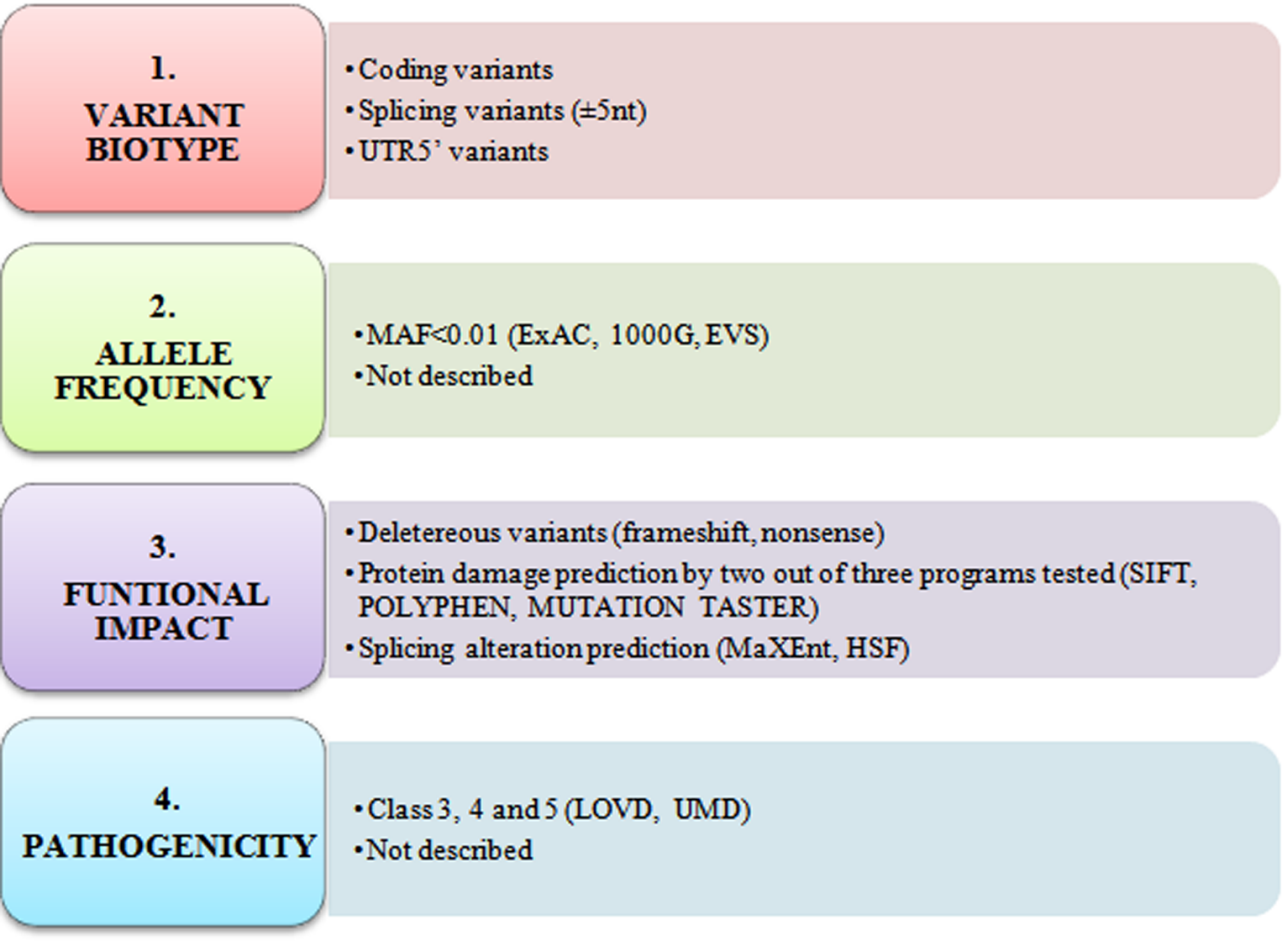
Filtering strategy for the detection of candidate variants.

The filtering strategy is described in Fig 1. All rare (novel or MAF<0.01), deleterious or possibly deleterious variants (according to protein and/or splicing alteration prediction tools) were selected for validation by direct sequencing. MaxEnt and HSF [11] were used to predict splicing alterations and SIFT [12], Polyphen2 [13] and MutationTaster [14] to predict protein damage. *GALNT12* candidate variants have been submitted to ClinVar, NCBI (https://www.ncbi.nlm.nih.gov/clinvar/).

### Segregation and LOH

Both loss of heterozygosity (LOH) in adenoma/tumor DNA and segregation analyses in the germline DNA of family members were achieved by direct sequencing.

### FFPE-immunohistochemistry

Immunohistochemistry of total and unglycosylated MUC1 was performed on 5 formalin-fixed, paraffin-embedded (FFPE) matched normal and adenoma tissue sections. MUC1 (VU4H5) mouse monoclonal antibody (Cell Signaling Technology, U.S.), which binds to the extracellular domain only if the threonine residue of the tandem repeat is not glycosylated, was used to specifically detect non-glycosylated MUC1. MUC1 (EMA, E29) mouse monoclonal antibody (DAKO, Thermo Fisher Scientific, U.S.), which reacts with the MUC1 cytoplasmic domain, was used to detect total MUC1. Detection was done with the Dako Omnis Staining System (DAKO) and slides were counterstained with hematoxylin. Two expert pathologists reviewed all the staining. Levels of staining were classified into 0, +, ++, +++ according to the antibody intensity.

### Case-control study

Germline DNAs from 451 unrelated polyposis cases and 714 cancer-free controls were tested for c.907G>A, p.(D303N) by TaqMan genotyping assays (Thermo Fisher Scientific, U.S.). Data from the 1000 Genomes Project [15], Exome Variant Server [16] and Exome Aggregation Consortium [17] was also used to check the association of c.907G>A, p.(D303N) with the AAP cohort. Fisher’s exact test was used to assess differences in the allelic distribution between cohorts and CaTS [18] to estimate the statistical power. A ρ value of 0.05 was considered statistically significant.

## Results and discussion

Summary of the clinicopathological characteristics of the study and validation cohorts are shown in Table 2.

**Table 2:** Clinicopathological characteristics of study cohorts.

	AAP COHORT	VALIDATION COHORT
N	183	268
Dx Age; average(range)	61 (32–79)	64.3 (30–85)
N adenoma; average (range)	30 (10–100)	20 (10–150)
Hyperplastic polyps	87 (47.5%)	60 (22.5%)
Full-blown AAP^a^	140 (76,5%)	164 (61.2%)
CRC	49 (26.7%)	78 (29.1%)

^a^Full-blown polyposis = subjects with more than 20 adenomas or 10 synchronic adenomas. The remaining subjects presented between 10–20 adenomas.

Germline DNAs from 183 AAP patients (Table 2) were screened for mutations in the *GALNT12* gene. Identified variants are shown in S1 Table. After applying our filtering strategy (Fig 1) only 4 cases were found to harbor the *GALNT12* variant c.907G>A, p.(D303N) and no other candidate *GALNT12* variants were detected (S1 Table). c.907G>A, p.(D303N) carriers were all screened by the 22-gene NGS panel, therefore carrier data from the remaining genes was reviewed and filtered according to the same strategy, and no other candidate variants were found in any other CRC susceptibility gene (S2 Table). All probands harboring c.907G>A (p.D303N) presented full-blown late-onset AAP, and all of them had both adenoma and CRC family history (Table 3).

**Table 3:** Clinical characteristics of c.907G>A, p.(D303N) carriers.

Family	Id	Inclusion criteria	Dx age^a^	No. of adenomas	Cancer	
					type	age
**PAX1**	I:1	20A^b^	71	>50	no	
**PAX2**	I:1	10SA^c^	80	12	no	
**PAX3**	I:1	20A^b^	50	>30	no	
**PAX4**	I:1	20A^b^	78	37	CRC/renal	78/76

^a^Dx age: age at diagnosis of polyposis.

^b^20A = more than 20 adenomas

^c^10SA = 10 synchronic adenomas.

Looking at public genome databases, c.907G>A, p.(D303N) is a rare allele present in less than 0.3% of all tested populations, which makes a statistical difference with respect to the polyposis study cohort (Table 4). Moreover, as it has been mentioned above, c.907G>A, p.(D303N) had been previously described in CRC patients, functional characterization of the encoded protein showed a partial activity of 37% when compared with the wild type protein [4] and co-segregation with CRC and polyps was also described in families fulfilling the Bethesda clinical criteria [5]. Together, all this data suggest that the c.907G>A, p.(D303N) variant could be a quite good high-predisposition candidate allele for unexplained AAP. Therefore, subsequent tests were performed in order to shed light on the polyposis causality of the c.907G>A, p.(D303N) variant. For this purpose, segregation and LOH analyses, glycosylation pattern tests and case-control studies were achieved.

**Table 4:** c.907G>A, p.(D303N) allelic population frequencies and association analysis.

POPULATION	N	ALT^g^	REF^h^	MAF	ρ-value	OR
**AAP**^**a**^	**183**	**4**	**362**	**0,011**		
1000G^b^	503	3	1003	0.003	0.087	3.69
EVS^c^	4300	1	8589	0.001	0.003	8.63
ExAC^d^	31072	122	62022	0.002	0.007	5.62
HCSC^e^	714	4	1424	0.003	0.059	3.93
**VAL**^**f**^	**268**	**0**	**536**	**0**		
HCSC	714	4	1424	0.003	0.41	0.2950
**AAP+VAL**	**451**	**4**	**898**	**0,004**		
HCSC	714	4	1424	0.003	0.496	1.59

^a^AAP = attenuated adenomatous polyposis study cohort.

^b^1000G = European population from the 1000 Genomes database.

^c^EVS = European-American population from the Exome Variant Server.

^d^ExAC = Non-Finnish European population from the Exome Aggregation Consortium database.

^e^HCSC = cancer-free control population recruited at Hospital Clínico San Carlos (Madrid).

^f^VAL = validation cohort.

^g^ALT = altered allele count

^h^REF = reference allele count.

**Fig 2:**
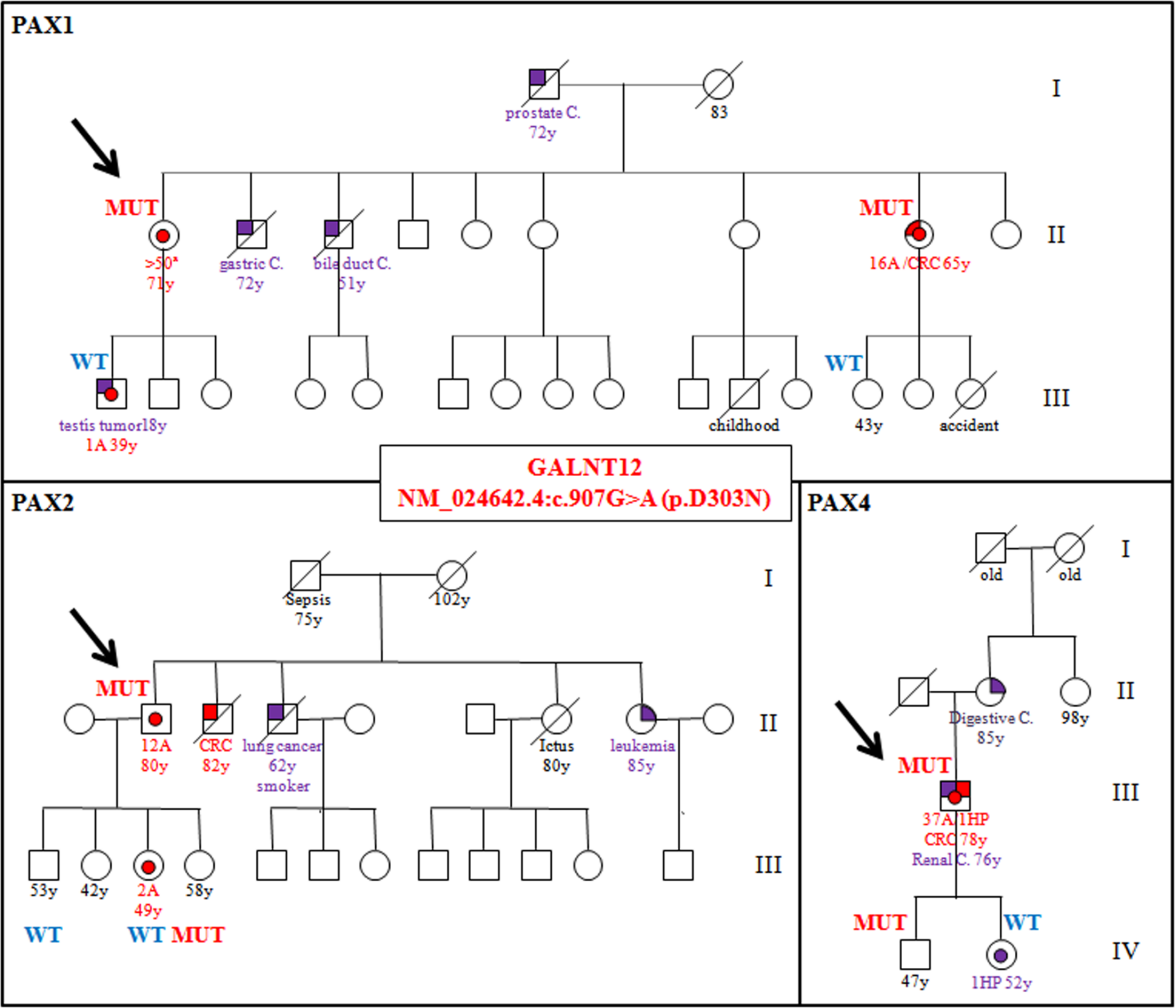
Pedigrees of families harboring c.907G>A (p.D303N) and segregation analysis. Probands are marked with arrows. MUT = individual carrying the c.907G>A (p.D303N) allele. WT = individual not carrying the c.907G>A, p.(D303N) allele. CRC = colorectal cancer. y = age at diagnosis or age at the time of DNA extraction (in healthy subjects).

First, the variant was tested in the germline DNA of those relatives who had agreed to participate in the study. None of the three families tested for segregation showed a clear high-penetrance allele segregation pattern (Fig 2). There was only one relative with polyposis diagnosis (pedigree PAX1_II:8) and she harbored the variant. In pedigrees PAX1 and PAX2, there were two relatives with a diagnosis of adenomas and they didn’t harbor the variant. Conversely, two relatives in pedigrees PAX2 and PAX4 harbored the variant and they didn’t show any adenoma at the ages of 58 and 47.

Since all probands showed late-onset AAP and most of the healthy relatives tested were in their forties, carriers who had not developed polyposis could still develop it at later ages. However, it is noteworthy that 2 relatives had already developed some adenomas at younger ages (Fig 2: PAX1_III:1 and PAX2_III:3) and none of them harbored the c.907G>A (p.D303N) variant. Described frequencies of adenomas in populations ranging from 40 to 50 years is around 4% [19, 20] so these cases could be explained as phenocopy phenomena, but within a context of suspected polyposis genetic predisposition we would not expect to have many cases of adenomas at early ages that are not explained by the putative polyposis susceptibility cause. Clarke et al. also observed similar results in two Bethesda families harboring the variant [5]. They observed two polyp cases and one CRC in which the variant was not detected. Therefore, considering both studies, co-segregation results are inconclusive.

Guda et al. [4] suggest that CRC associated *GALNT12* complete inactivation alleles are most likely to act as simple null allele rather than dominant effect or oncogenic alleles. Consequently, LOH events were investigated in 3 low-grade dysplastic adenomas, 2 high-grade dysplastic adenomas and 1 CRC (Table 5). No LOH of the c.907G>A, p.(D303N) allele was detected in any of the samples. It was not possible, though, to achieve full *GALNT12* somatic mutational screening and methylation analysis, so we cannot discard other somatic second hits at *GALNT12* or haplosinsuffiency mechanisms leading to the adenoma development. In any case, we would expect a decrease in GALNT12’s activity and therefore to an increase in improperly glycosylated or unglycosylated proteins in carrier adenomas. In order to test this, two antibodies against both, total and unglycosylated forms of GALNT12’s target protein MUC1, were tested to detect any change in the glycosylation pattern of the same 5 adenomas tested for LOH and their matched normal tissues similarly to Guda et al. [4]. Any alteration in the glycosylation activity would imply an increase in the amounts of unglycosylated but not total MUC1 levels. However, no differences were detected in any of the adenoma-normal tissue pairs (Table 5 and S1 Fig), ruling out the causality of this allele in the adenoma development.

**Table 5:** Adenomas tested for LOH and MUC1 IHC.

Family	Patient	Adenoma	Histology	LOH	IHC-N^a^	IHC-T^b^
PAX1	I:1	12B3204	Low-grade tubulovillous adenoma	NO	++/+++	++/+++
		13B12094	Low-grade tubular adenoma	NO	++/+++	++/+++
PAX2	I:1	9B66481	High-grade tubular adenoma	NO	++/+++	++/+++
		9B66483	Low-grade tubular adenoma	NO	++/+++	++/+++
PAX3	I:1	16B23342	High-grade tubular adenoma	NO	++/+++	++/+++
		15B10052	Adenocarcinoma Haggitt 4	NO		

^a^IHC-N = IHC evaluation for matched-normal tissues (ratio between non-glycosylated MUC1 and total MUC1 levels)

^b^IHC-T = Immunochemistry evaluation for matched-adenomatous tissue (ratio between non-glycosylated MUC1 and total MUC1 levels).

Taken together, these results make us distrust c.907G>A, p.(D303N) as a high-predisposition allele for AAP. Nevertheless, due to the high association of the variant with the study cohort and in order to validate the previous results, a case-control study was conducted in a larger validation cohort with the same inclusion criteria (Table 2) and a home cancer-free control cohort. In this case, the statistical analysis did not detect significant differences on the c.907G>A, p.(D303N) allele frequency between cohorts (Table 4). The clinical information about the 4 controls harboring the c.907G>A, p.(D303N) allele was further investigated. Two of them died at old age without any personal or familial history of adenomas. One other control had two second-degree relatives with late onset CRC (>70), but underwent several colonoscopies with no adenomas detected. And the last one had a melanoma diagnosis at the age of 42.

It should be noted, though, that due to the low frequency of the variant in control populations, the sample size used in this analysis allow us to detect statistical significance with a minimum statistical power of 80% when the OR is greater than 4. Therefore, the case-control analysis discards the c.907G>A, p.(D303N) variant as a high-penetrance risk allele for AAP, but not as a low-penetrance allele. Our results discard the causality of c.907G>A, p.(D303N) in hereditary polyposis CRC syndromes but not the role of the variant as a modifier allele or its involvement in other forms of sporadic polyposis, which points to the necessity of larger sample sizes to be able to further confirm or discard the role of this variant as a low-penetrance allele.

These results, together with the absence of other functionally relevant *GALNT12* mutations in our cohort, discard *GALNT12* as a major high-predisposition gene for adenomatous polyposis. Considering that its major role as a high-predisposition allele for HNPCC had been previously discarded [6], its major role in hereditary CRC syndromes can be ruled out.

With the development of next-generation sequencing technologies and their integration in diagnosis laboratories, the screening of candidate high-predisposition genes that have been proposed, but not validated, is an attractive but very dangerous tool for the understanding of those unexplained familial cancer cases. Hence, the elucidation of cancer-risk candidate genes and variants becomes essential to avoid clinical misinterpretation of cancer risk estimations in carrier families. Here we show how an initially good high-risk candidate variant for polyposis predisposition is finally dismissed after complementary assays. Finally, the role of *GALNT12* as a major high-predisposition gene for inherited CRC is not confirmed, and the inclusion of *GALNT12* in the screening of high-penetrance alleles in unexplained familial CRC is discouraged.

## Supporting information

S1 Table *GALNT12* variants detected in the AAP population. (PDF)

S2 Table Rare variants in other CRC susceptibility genes detected in *GALNT12*_c.907G>A, p.(D303N) carriers. (PDF)

S1 Fig Unglycosylated and total MUC1 detection in the *GALNT12*_c.907G>A, p.(D303N) carrier adenomatous and normal matched tissues. (PDF)
