# Supplementary material for: Role of GALNT12 in the genetic predisposition to attenuated adenomatous polyposis syndrome
Source: PLoS One. 2017 Nov 2;12(11):e0187312. doi: 10.1371/journal.pone.0187312 (PMC5667827; doi:10.1371/journal.pone.0187312)
Supplement: S1 Table — (PDF) [file pone.0187312.s001.pdf]

S1 Table. *GALNT12* variants detected in the AAP population. Only c.907G>A (p.D303N) was selected after applying the filtering strategy (red labelled).

CHR POSITION	HGVS[a]	rs[b]	ExAC[c]	EVS[d]	1000G[e]	PD[f]	SA[g]
9:101570336	c.356A>T; p.E119V	rs10987769	0.177	0.079	0.086	2	NO
9:101585643	c.477C>G; p.V159=		0	0	0	na	NO
9:101589058	c.566A>G; p.N189S	rs183981750	0.0007	0.0002	0	0	NO
9:101594103	c.781G>A; p.D261N	rs41306504	0.0125	0.0114	0.01	2	NO
**9:101594229**	**c.907G>A; p.D303N**	**rs145236923**	**0.0019**	**0.0013**	**0.003**	**2**	**NO**
9:101594263	c.917+24C>T	rs41297187	0.0301	0.0229	0.019	na	NO
9:101599421	c.1203T>G; p.R401=	rs140977555	0	0	0	na	NO
9:101606425	c.1392C>G; p.P464=	rs35616709	0.0035	0.0059	0.003	na	NO
9:101611335	c.1707G>C p.S569=	rs2273846	0.0606	0.0521	0.069	na	NO
9:101611545	c.*171A>G	rs2273847	-	-	0.069	na	na
9:101611697	c.*323A>C	rs573046827	-	-	0.001	na	na
9:101611795	c.*421G>A	rs2273848	-	-	0.047	na	na

[a]HGVS= HGVS variant designation according to the transcript NM_024642 and the protein NP_078918. [b]rs= reference SNP ID. [c]ExAC= MAF for Non-Finnish European population from the Exome Aggregation Consortium database. [d]EVS= MAF for the European-American population from the Exome Variant Server. [e]1000G= MAF for the European population from the 1000 Genomes database. [f]PD= number of programs with protein damage prediction among the three programs tested (SIFT, Polyphen2 and MutationTaster), na=not applicable. [g]SA= splicing alteration prediction according to HSF and MaxEnt algorithms; NO= no alteration prediction, na=not applicable.
