# Supplementary material for: Role of GALNT12 in the genetic predisposition to attenuated adenomatous polyposis syndrome
Source: PLoS One. 2017 Nov 2;12(11):e0187312. doi: 10.1371/journal.pone.0187312 (PMC5667827; doi:10.1371/journal.pone.0187312)
Supplement: S2 Table — (PDF) [file pone.0187312.s002.pdf]

S2 Table. Rare variants in other CRC susceptibility genes detected in *GALNT12*_c.907G>A (p.D303N) carriers. Only those variants with MAF<0.01 are shown.

CHR POSITION	GENE	HGVS[a]	rs[b]	ExAC[c]	EVS[d]	1000G[e]	PD[f]	SA[g]
19:50905096	**POLD1**	c.378C>T; p.R126=	rs145324823	0,0018	0,0021	0,002	na	NO
19:50912169	**POLD1**	c.1892+11C>T	rs376751542	0	0,00012	0,0046	na	NO
2:47643476	**MSH2**	c.786C>T; p.A262=	rs4987189	0,0017	0,0014	0.0078	na	NO
5:79965999	**MSH3**	c.663T>C; p.A221=		0	0	0	na	NO

[a]HGVS= HGVS variant designation according to the transcripts POLD1: NM_001256849, MSH2: NM_001258281, MSH3: NM_002439 and the protein POLD1: NP_001243778, MSH2: NP_001245210, MSH3: NP_002430. [b]rs= reference SNP ID. [c]ExAC= MAF for Non-Finnish European population from the Exome Aggregation Consortium database. [d]EVS= MAF for the European-American population from the Exome Variant Server. [e]1000G= MAF for European population from the 1000 Genomes database. [f]PD number of programs with protein damage prediction among the three programs tested (SIFT, Polyphen2 and MutationTaster), na=not applicable. [g]SA= splicing alteration prediction according to HSF and MaxEnt algorithms; NO= no alteration prediction.
