# Supplementary material for: Role of GALNT12 in the genetic predisposition to attenuated adenomatous polyposis syndrome
Source: PLoS One. 2017 Nov 2;12(11):e0187312. doi: 10.1371/journal.pone.0187312 (PMC5667827; doi:10.1371/journal.pone.0187312)
Supplement: S1 Fig — (PDF) [file pone.0187312.s003.pdf]

S1Fig. Unglycosylated and total MUC1 detection in the *GALNT12*_c.907G>A (p.D303N) carrier adenomatous and normal matched tissues.

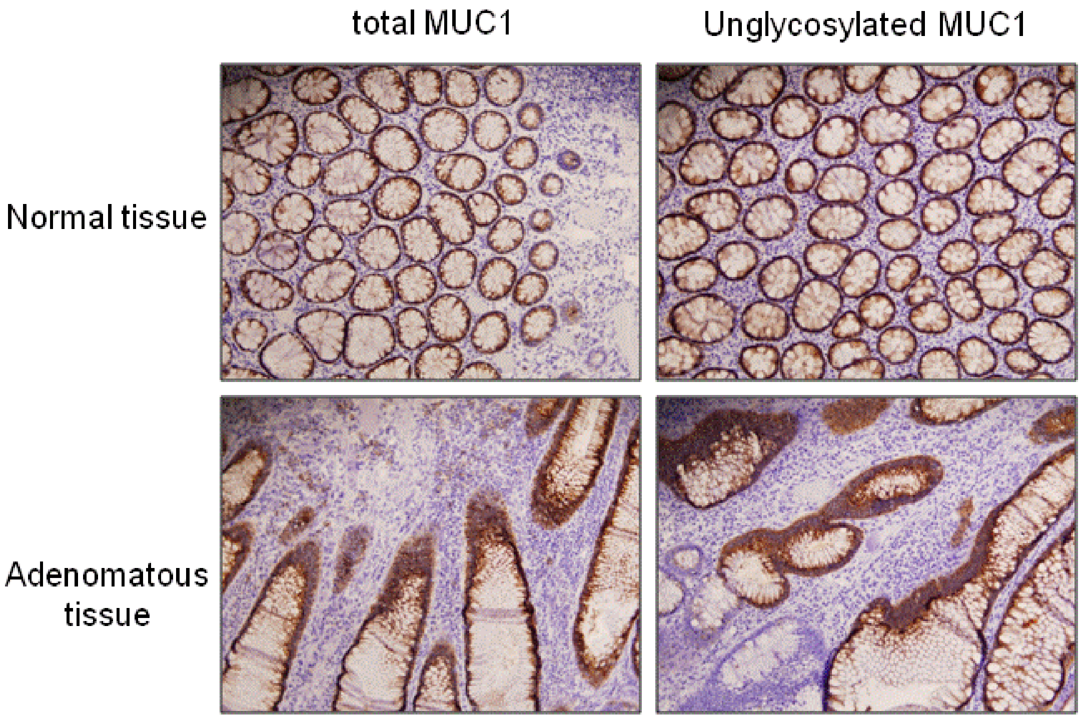

Immunohistochemistry of total and unglycosylated MUC1 was performed on formalin-fixed, paraffin-embedded (FFPE) matched normal and adenoma tissue sections. MUC1 (VU4H5) mouse monoclonal antibody (Cell Signaling Technology) was used to specifically detect non-glycosylated MUC1, and MUC1 (EMA, E29) monoclonal antibody (DAKO) was used to detect total MUC1. Detection was done with the Dako Omnis Staining System (DAKO) and slides were counterstained with hematoxylin.
